# Interictal cytokine levels were correlated to seizure severity of epileptic patients: a retrospective study on 1218 epileptic patients

**DOI:** 10.1186/s12967-015-0742-3

**Published:** 2015-12-01

**Authors:** Ye Wang, Desheng Wang, Dawen Guo

**Affiliations:** Department of Neurology, First Affiliated Hospital of Harbin Medical University, Harbin, Heilongjiang 150001 Peoples’ Republic of China; Department of Clinical Laboratory, First Affiliated Hospital of Harbin Medical University, Harbin, Heilongjiang 150001 Peoples’ Republic of China

**Keywords:** Biomarker, Cytokine, Epilepsy, Seizure, Disease severity

## Abstract

**Background:**

Many aspects on the correlation between epilepsy and cytokine levels were unclear. This study aims to investigate the correlations between cytokine levels and severe epilepsy.

**Methods:**

Totally 1218 epileptic patients were grouped by types of epilepsy: TLE (temporal lobe epilepsy, n = 409), XLE (extra-temporal lobe epilepsy, n = 290) and IGE (idiopathic generalized epilepsy, n = 519). Two hundred healthy volunteers were as controls. Clinical findings and levels of 14 serum and CSF cytokines and 6 STAT members were collected, measured and analyzed.

**Results:**

Analysis showed no differences in interictal cytokine levels among patients from TLE, XLE and IGE groups. Interictal serum levels of IL-1b, IL-1Ra, IL-6, IL-8, IFNγ, IFNλ3 and IL-17a were associated with seizure severity of epileptic patients, measured by seizure frequency, VA score or NHS3. Multivariate regression analysis indicated that interictal concentrations of serum IL-6, IFNγ, IL-17a, IFNλ3, and CSF IL-6, IL-17a, IFNλ3 were significant biomarkers for patients with severe epilepsy. mRNA levels of IL-6, IFNγ, IL-17a, and IFNλ3 were elevated in different types of epilepsy. Activation of all STATs was elevated in epilepsy, and STAT3 was activated 9-fold in average, which was the highest among all STATs.

**Conclusions:**

Interictal serum IL-6, IFNγ, IL-17a, IFNλ3, and CSF IL-6, IL-17a, IFNλ3 could be used as potential biomarkers for severe epilepsy. Activation of STATs, especially STAT3, was important in epilepsy. Our findings pointed out crucial roles of cytokine levels in epilepsy.

## Background

Cytokines and chemokines are important mediators in many physiological and pathological modulations including nervous system development, bidirectional signal transduction between central and peripheral nerve systems, cognitive processes, and etc. [1, 2]. Under most physiological conditions, cytokine levels are low, but they could increase manifold up to hundreds of times their basal concentrations in pathological conditions. It has been reported that adult brain cells express cytokines such as IL-1b, IL-6, IL-8, IL-10, IL-12, IL-15, TNF, CCL2, CCL3 and CCL4 [3]. New cytokines are being identified in CNS (central nerve system).

Regulations of expression and secretion of cytokines and their receptors have been described in patients with epilepsy, in addition to animal models of epilepsy [4, 5]. For example, serum IL-1b, IL-1Ra, IL-2, IL-4, IL-6, IL-8, IFNγ, and IL-17 concentrations were observed to be elevated in patients with epilepsy [4–8]. Elevated IL-6 and IL-17 levels in CSF (cerebrospinal fluid) were also reported in publications [8–10]. In addition, both IL-6 and CCL2 are elevated in the temporal cortex of pediatric patients from families with epilepsy history [11]. A recent case–control study indicated that after blocking IL-6R with the monoclonal antibody, tocilizumab, stable remission of epileptic symptoms could be achieved [12]. Additional reports showed that IL-4, IL-8 and IL-17 concentrations may be correlated to seizure frequency and severity [7–9, 13]. All above evidences suggest key roles of cytokines in diagnosis and treatment of epilepsy. But the definite correlations between above cytokines and different types of epilepsy, and with seizure severity still need to be investigated.

The objective of this study was to analyze the clinical and laboratory data of a group of epileptic patients, and evaluate the correlations between interictal cytokine concentrations and seizure severity in different types of epilepsy. This study aims to identify serum markers of disease severity in order to facilitate more accurate diagnosis of severe epilepsy, so that prophylactic measures can be taken.

## Methods

### Study population

Our study population consisted of 1218 interictal patients with symptomatic epilepsy who were treated at our institution from January 2009 to February 2015. Exclusion criteria include a history of autoimmune diseases, allergic response, immune deficiency disorder, diabetes, psychiatric illness, malignancy, severe cognitive impairment, or a systemic or central nervous system (CNS) infection 2 weeks before sample collection. Two hundred age- and sex-matched healthy volunteers were involved in this study.

Epilepsy was diagnosed by at least two licensed and experienced neurologists according to the 2006 International League Against Epilepsy (ILAE) Classification [14]. Seizure frequency was evaluated by using seizure diaries and seizure severity was by using the National Hospital Seizure Severity Scale (NHS3) and the Veterans Administration Seizures Frequency and Severity Rating Scale score (VA score) [15]. TLE (temporal lobe epilepsy), XLE (extra-temporal lobe epilepsy) and IGE (idiopathic generalized epilepsy) were diagnosed based on medical history, electro-clinical findings (including seizure semiology and EEG/video-EEG) and neuro-imaging [10]. A high-resolution 1.5 Tesla magnetic resonance imaging (MRI) scan of the brain with a specific epilepsy protocol was obtained to define the etiology, which was classified as normal and abnormal.

### Data source

All of the interictal parameters included in the investigation were collected at day 7 from the last seizure attack. The collected clinical parameters included demographic characteristics (age and sex), clinical parameters (signs and symptoms), laboratory values (hematologic, biochemical and microbiological findings), radiologic data, epileptic duration, seizure frequency per month in the past year, and number of epileptic drugs.

The Harbin Medical University First Hospital Ethics Committee approved the study, and all involved healthy volunteers and patients gave written informed consent for their clinical data and samples (blood, serum and CSF) to be used in this study. Human experimentation guidelines of PR.China were followed in the conduct of this research.

### Cytokine level determination

Interictal samples were collected at day 7 from the last seizure attack. The plasma was harvested within 30 min at 37 °C of venipuncture from EDTA-anticoagulated blood samples and stored at −80 °C for subsequent cytokine analysis. The concentrations of IL-2, IL-4, IL-6, IL-8, IL-10, IFNγ, GM-CSF, TNFα (Bio-Rad, USA), IL-17a (PeproTech, Rocky Hill, NJ, USA), IL-1β (Bender MedSystems, Vienna, Austria), IL1Ra (Cytoscreen, Biosource, Belgium), IFNλ1, IFNλ2, IFNλ3, IFNλ4 (eBioscience, CA, USA), and IL-23 (Invitrogen, Carlsbad, CA, USA) were measured by ELISA according to manufacturers’ instruction [16–20]. CSF concentrations of IL-6, IFNγ, IFNλ3 and IL-17a were measured using human cytoline/chemokine MILLIplex kits (Millipore Corp, Billerica, MA, USA) [21]. ELISAs were performed in duplicate.

### Statistical analysis

Averages of numerical variables were presented as $\bar{\chi }$ ± SD. Differences were compared by Chi square test for categorical data and unpaired Student’s *t* test for continuous normally distributed data (tested by Wilk-Shapiro test). Cytokine concentrations were compared by Mann–Whitney U test. Correlation between cytokine levels and seizure frequency/severity was analyzed by Spearman correlation and multivariate linear regression analysis. In multivariate regression analysis, variables with a p-value less than 0.05 were included in the model, and all of the continuous data had skewed distribution and were logarithmically transformed to fit normal distribution. All analyses were performed by SPSS software (version 11.0). Statistical data in multivariate regression analysis were shown with P value <0.05 or <0.01 to be considered as statistically significant.

## Results

### Patient characteristics

The demographic and clinical data of 1218 patients with different types of epilepsy were collected and summarized in Table 1. The average duration of their hospital stay was 13.77 ± 11.22 days. All patients were recovered and discharged, and no one died. The epileptic patients involved in this study were categorized into three groups (TLE (n = 409), XLE (n = 290), and IGE (n = 519)). Except for brain MRI, there were no statistic differences between groups in term of clinical characteristics (all P values >0.05, Table 1). Laboratory findings of epileptic patients in each study group were shown in Table 2. No significant differences were found between groups (all P values >0.05).

**Table 1:** Clinical characteristic of epileptic patients

Epilepsy syndrome	TLE	XLE	IGE
n	409	290	519
Female/male	90/319	51/239	110/409
Age (mean ± SD, years)	33.2 ± 12.4	31.0 ± 10.9	30.8 ± 10.8
Epilepsy duration (mean ± SD, years)	15.1 ± 9.8	19.2 ± 15.2	10.9 ± 11.2
Seizure frequency (mean ± SD, seizure/month)	10.0 ± 12.4	19.3 ± 17.3	15.5 ± 11.3
Brain MRI (n, %)			
Normal	316 (77.3)	190(65.5)	519 (100)
Abnormal	93 (22.7)	100 (34.5)	0
Side of epilepsy (n, %)			
Left	119 (29.1)	78 (26.9)	136 (26.2)
Right	192 (46.9)	76 (26.2)	166 (32.0)
Unidentified	98 (24.0)	136 (46.9)	217 (41.8)
Anti-epileptic drugs (n, %)			
None	7 (1.7)	5 (1.7)	7 (1.4)
Mono-drug	37 (9.0)	39 (13.4)	76 (14.7)
Poly-drug	365 (89.2)	246 (84.9)	436 (84.0)
Refractory epilepsy (n, %)	377 (92.2)	268 (92.4)	468 (90.1)

Refractory epilepsy was defined as >2 seizures/month during last year under pharmacologically treatment

*VA score* Veterans Administration Seizures Frequency and Severity Rating Scale score, *NHS3* National Hospital Seizure Severity Scale, *TLE* temporal lobe epilepsy, *XLE* extra-temporal lobe epilepsy, *IGE* idiopathic generalized epilepsy

**Table 2:** Laboratory findings

	TLE (n = 409)	XLE (n = 290)	IGE (n = 519)
GLU (mmol/L)	7.3 ± 2.4	6.9 ± 3.3	7.6 ± 4.1
WBC (10^9^/L)	9.3 ± 4.5	10.4 ± 3.2	9.2 ± 4.7
Neutrophils (%)	57.7 ± 21.6	57.1 ± 22.8	60.5 ± 25.7
Lymphocytes (%)	39.6 ± 15.6	37.2 ± 20.5	40.0 ± 16.3
HB (g/dL)	132.2 ± 22.3	118.9 ± 32.1	119.4 ± 33.0
PLT (10^9^/L)	301.2 ± 103.4	290.1 ± 111.1	288.9 ± 143.2
ALT (IU/L)	29.6 ± 25.3	21.2 ± 31.7	27.7 ± 25.7
AST (IU/L)	32.5 ± 26.2	34.9 ± 28.2	31.5 ± 26.5
CK-MB (IU/L)	17.2 ± 28.3	19.3 ± 23.7	17.3 ± 31.1
CRP (mg/L)	3.2 ± 4.2	3.7 ± 4.9	3.3 ± 3.4
LDH (U/L)	254.1 ± 73.2	253.2 ± 76.4	243.6 ± 70.0
K (mmol/L)	4.1 ± 1.3	4.1 ± 1.8	4.3 ± 1.9
Na (mmol/L)	134.8 ± 3.8	135.3 ± 3.3	136.2 ± 4.0
CL (mmol/L)	102.3 ± 39.8	109.2 ± 38.0	101.3 ± 38.3

*GLU* blood glucose, *LYM* percentage of lymphocytes, *ALT* alanine aminotransferase, *CL* blood chlorine, *WBC* white blood cell counts, *CK* creatine kinase, *CK-MB* creatine kinase-MB, *CRP* C-reactive protein, *LDH* Lactate dehydrogenase, *TLE* temporal lobe epilepsy, *XLE* extra-temporal lobe epilepsy, *IGE* idiopathic generalized epilepsy

### Correlation between interictal cytokine levels and different types of epilepsy

To discover potential serum biomarkers for differentiate TLE, XLE and IGE, we tested 14 interictal cytokine concentrations (IL-1b, IL-1Ra, IL-2, IL-4, IL-6, IL-8, IL-10, IFNγ, IL-17a, IFNλ1, IFNλ2, IFNλ3, IFNλ4, and IL-23). The levels of tested cytokines in patients with different types of epilepsy were analyzed. Unfortunately, none of these cytokine concentrations showed any statistical significance between different epileptic groups (all P > 0.05).

### Correlation between interictal serum cytokine levels and seizure severity

Next, we tried to investigate whether interictal cytokine levels could be used as markers to indicate seizure severity in patients with different types of epilepsy. Seizure frequency, VA score, and NHS3 were used as seizure severity scales. Table 3 demonstrated statistical analysis results on each cytokine. If seizure frequency was used for seizure severity, then levels of IL-6, IL-8 and IL-17a were significant biomarkers in all three types of epilepsy. IL-1Ra was for TLE and XLE (P = 0.041 and 0.032, respectively), IFNγ, IFNλ2 and IFNλ4 were for XLE (P = 0.008, 0.004 and 0.022), and IFNλ3 for IGE (P = 0.003). If VA score was applied, IL-1Ra, IL-6, IFNγ, IFNλ3 and IL-17a were severity markers in all types of epilepsy. IFNλ2 and IFNλ3 were for TLE (P = 0.042 and 0.008). IL-1b, IL-8, IFNλ1 and IFNλ3 were for XLE (P = 0.033, 0.005, 0.006, and 0.010 respectively). If NHS3 was used, IL-6, IFNγ and IFNλ3 were good markers in all types of epilepsy. IL-1Ra, IL-8 and IL-17a were for TLE (P = 0.020, 0.012 and 0.021, respectively), IL-17a and all IFNλs were for XLE (P = 0.049, 0.025, 0.049, <0.001 and 0.006), and IL-1Ra, IL-8, IFNλ1, and IFNλ4 were for IGE (P = 0.009, 0.005, 0.011 and 0.043 respectively). Among all cytokine concentrations, only IL-6 level was correlated to all three severity scaling systems in all three types of epilepsy. IFNλ3 was also good for all scaling systems in all types of epilepsy, except for frequency in TLE and XLE.

**Table 3:** Correlation analysis between cytokine levels and disease severity in different types of epilepsy

	TLE (n = 409)			XLE (n = 290)			IGE (n = 519)		
	Freq	VA	NHS3	Freq	VA	NHS3	Freq	VA	NHS3
IL-1b	0.058	0.098	0.075	0.069	0.033*	0.141	0.069	0.091	0.143
IL-1Ra	0.041*	0.004**	0.020*	0.032*	0.021*	0.078	0.059	0.005**	0.009**
IL-2	0.095	0.121	0.119	0.077	0.089	0.083	0.132	0.244	0.097
IL-4	0.249	0.192	0.104	0.193	0.087	0.102	0.201	0.167	0.131
IL-6	0.002**	0.045*	0.005**	0.003**	0.042*	<0.001**	0.001**	<0.001**	0.003**
IL-8	0.007**	0.077	0.012*	0.001**	0.005**	0.083	0.031*	0.051	0.005**
IL-10	0.210	0.088	0.089	0.192	0.094	0.078	0.201	0.194	0.076
IFNγ	0.081	0.011*	0.008**	0.008**	0.004**	0.034*	0.066	0.018*	0.010*
IFNλ1	0.091	0.112	0.077	0.004**	0.006**	0.025*	0.092	0.082	0.011*
IFNλ2	0.022*	0.042*	0.121	0.394	0.112	0.049*	0.079	0.099	0.245
IFNλ3	0.101	0.008**	<0.001**	0.058	0.010*	<0.001**	0.003**	0.045*	0.004**
IFNλ4	0.100	0.192	0.492	0.022*	0.051	0.006**	0.148	0.051	0.043*
IL-17a	0.001**	<0.001**	0.021*	0.023*	0.011*	0.049*	0.036*	0.001**	0.078
IL-23	0.172	0.099	0.221	0.255	0.301	0.175	0.141	0.187	0.251

*Freq* seizure frequency per month, *VA score* Veterans Administration Seizures Frequency and Severity Rating Scale score, *NHS3* National Hospital Seizure Severity Scale, *TLE* temporal lobe epilepsy, *XLE* extra-temporal lobe epilepsy, *IGE* idiopathic generalized epilepsy

* P < 0.05, ** P < 0.01

**Fig. 1:**
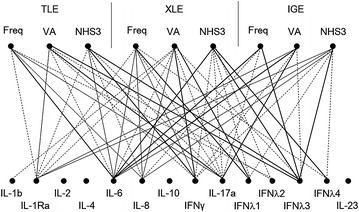
Correlation web of biomarkers and severity indices for three types of epilepsies. The cross-correlation of cytokine levels with seizure indices for three types of epilepsies was shown in the form of directed correlation web. The *line* thickness indicates the significance of the correlations. *Bold line* The statistical correlation is <0.05 in multivariate analysis; *Thin line* The statistical correlation is <0.01 in univariate analysis; Dash line: The statistical correlation is <0.01 in univariate analysis. *TLE* temporal lobe epilepsy, *XLE* extra-temporal lobe epilepsy, and *IGE* idiopathic generalized epilepsy

Furthermore, multivariate regression analysis revealed that interictal IL-6 concentration was positively related to seizure frequency in TLE and IGE (P = 0.041 and 0.002, respectively), VA score in IGE (P < 0.001), and NHS3 in XLE (P = 0.005). IFNγ level was positively linked to VA score in XLE (P = 0.015), and NHS3 in TLE (P = 0.023). IL-17a level was positively associated with VA score in TLE and IGE (P = 0.010 and 0.022, respectively). IFNλ3 concentration was positively linked to NHS3 in TLE and IGE (P = 0.019 and 0.022), and seizure frequency in IGE (P = 0.034) (Table 4). The overall data of statistical analysis on correlations between seizure severity and cytokine levels in TLE, XLE and IGE were shown graphically in Fig. 1. If P < 0.01 was considered significant, then only IL-6 concentration was positively linked to NHS3 in XLE (P = 0.005), seizure frequency and VA score in IGE (P = 0.002 and <0.001, respectively).

**Table 4:** Independent biomarkers for severe seizures

	TLE (n = 409)			XLE (n = 290)			IGE (n = 519)		
	Freq	VA	NHS3	Freq	VA	NHS3	Freq	VA	NHS3
IL-6	0.041					0.005*	0.002*	<0.001*	
IFNγ			0.023		0.015				
IFNλ3			0.019				0.034		0.022
IL-17a		0.010						0.022	

*Freq* seizure frequency per month, *VA score* Veterans Administration Seizures Frequency and Severity Rating Scale score, *NHS3* National Hospital Seizure Severity Scale, *TLE* temporal lobe epilepsy, *XLE* extra-temporal lobe epilepsy, *IGE* idiopathic generalized epilepsy

* P < 0.01

**Fig. 2:**
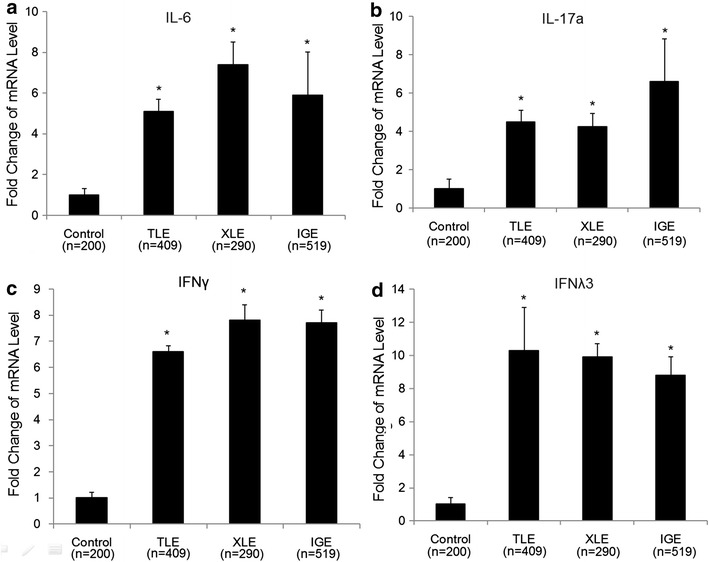
Serum mRNA concentration of IL-6, IL-17a, IFNγ and IFNλ3 were elevated in epilepsy. Serum mRNA concentration of IL-6 (**a**), IL-17a (**b**), IFNγ (**c**) and IFNλ3 (**d**) were measured in healthy controls and patients with TLE, XLE or IGE. Data represent a minimum of three independent experiments. Asterisks denote p < 0.05 in comparison to untreated samples. TLE: temporal lobe epilepsy, *XLE* extra-temporal lobe epilepsy, *IGE* idiopathic generalized epilepsy

The mRNA concentrations of 14 tested cytokines were evaluated, and the data on IL-6, IL-17a, IFNγ and IFNλ3 were shown in Fig. 2, since these four cytokines were identified in the multivariate analysis (Table 4). The mRNA levels of all these four cytokines were correlated to all three types of epilepsy. No difference was observed between types of epilepsy (all P > 0.05).

### Correlation between interictal cytokine levels and the time to next seizure episodes

Inflammation status may have an impact on the time to next seizure episodes, and cytokine levels are known biomarkers for inflammation intensity. So we analyzed the correlation between interictal serum cytokine levels and the time (days) to the next seizure episode since the study time point. All patients were followed up for 6 months. Data were shown in Table 5. IL-6 level was an independent biomarker for the time to next seizure episode in XLE (P = 0.002), IFNγ level was an independent biomarker in TLE (P = 0.041), IFNλ3 was in TLE and XLE (P = 0.002 and 0.032), and IL-17a was in TLE and XLE (P = 0.009 and <0.001, respectively). If P < 0.01 was considered statistically significant, then only IFNλ3 and IL-17a levels in TLE (P = 0.002 and 0.009, respectively), IL-6 and IL-17a in XLE (P = 0.002 and <0.001, respectively) were independent biomarkers.

**Table 5:** Correlations between interictal cytokine levels and the time to next seizure episode

	TLE (n = 409)		XLE (n = 290)		IGE (n = 519)	
	Univariate analysis	Multivariate analysis	Univariate analysis	Multivariate analysis	Univariate analysis	Multivariate analysis
IL-6	0.021	0.101	0.035	0.002*	0.007	0.067
IFNγ	0.002	0.041	<0.001	0.095	0.033	0.121
IFNλ3	0.019	0.002*	0.003	0.032	0.039	0.232
IL-17a	0.011	0.009*	0.002	<0.001*	0.032	0.112

*TLE* temporal lobe epilepsy, *XLE* extra-temporal lobe epilepsy, *IGE* idiopathic generalized epilepsy

* P < 0.01

### Correlation between interictal CSF cytokine levels and seizure severity

To further evaluate the association between cytokine levels and seizure severity, concentrations of CSF IL-6, IFNγ, IFNλ3 and IL-17a were tested on 150 patients selected from our different epileptic groups, respectively (50 of which were with TLE, XLE and IGE, respectively). These patients were randomly selected from the 1218 epileptic patients in this study, and their demographic and clinical features were matched between groups. Statistical analysis demonstrated that CSF IL-6 concentration was an independent markers for XLE if NHS3 was used for seizure severity (P = 0.007). CSF IL-17a was an independent marker for TLE and IGE if VA score was used (P = 0.039 and 0.044, respectively). CSF IFNλ3 was an independent marker for TLE and NHS3 if NHS3 was used (P = 0.022 and 0.012, respectively). If P < 0.01 was considered statistically significant, then only IL-6 in XLE could be recognized as an independent biomarker (Table 6). By univariate analysis, CSF IFNγ level was associated with TLE if VA or NHS3 (P = 0.045 and 0.033, respectively) was applied, but failed to enter the multivariate regression model.

**Table 6:** Interictal CSF IL-6, IL-17a, and IFNλ3 levels were independent biomarkers for seizure severity

	TLE (n = 50)			XLE (n = 50)			IGE (n = 50)		
	Freq	VA	NHS3	Freq	VA	NHS3	Freq	VA	NHS3
IL-6						0.007*			
IL-17a		0.039						0.044	
IFNλ3			0.022						0.012

*Freq* seizure frequency per month, *VA score* Veterans Administration Seizures Frequency and Severity Rating Scale score, *NHS3* National Hospital Seizure Severity Scale, *TLE* temporal lobe epilepsy, *XLE* extra-temporal lobe epilepsy, *IGE* idiopathic generalized epilepsy

* P < 0.01

### Activation of STAT family members was elevated in epilepsy

**Fig. 3:**
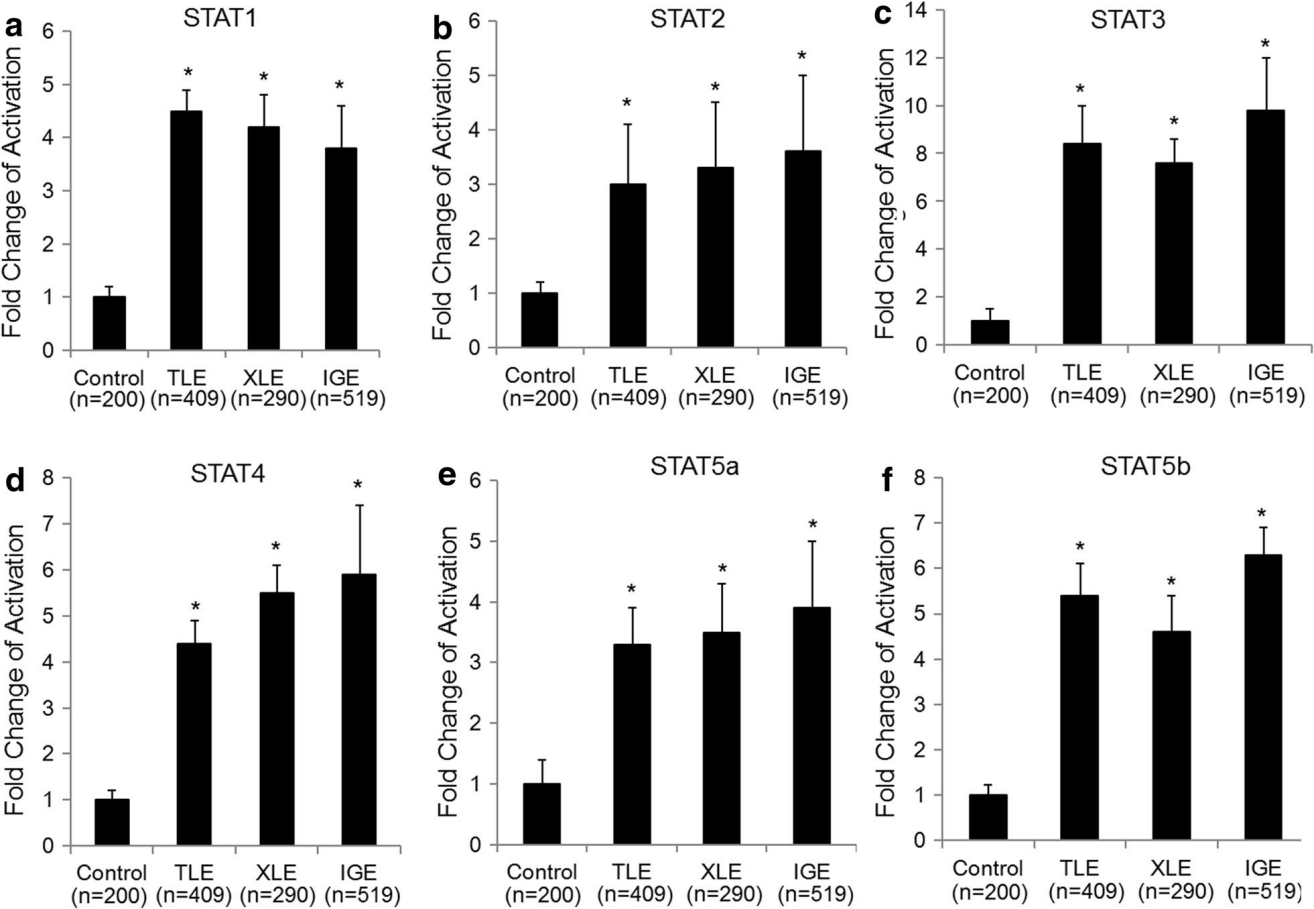
STATs were activated in patients with different types of epilepsy. PBMCs were extracted from healthy controls and different epileptic patients (TLE, XLE and IGE). Levels of activated STAT1 (**a**), STAT2 (**b**), STAT3 (**c**), STAT4 (**d**), STAT5a (**e**), and STAT5b (**f**) were measured by flow cytometry. Data represent a minimum of three independent experiments. Asterisks denote p < 0.05 in comparison to untreated samples. *TLE* temporal lobe epilepsy, *XLE* extra-temporal lobe epilepsy, *IGE* idiopathic generalized epilepsy

STATs are key signaling mediators induced by most interleukins and interferons [22]. The concentrations of activated all 6 STAT family members were evaluated in blood PBMCs of patients with different types of epilepsy. We found that activation of all tested STATs was significantly elevated in all three types of epilepsy. Among all STAT members, STAT3 activation level was increased ninefold in average, much higher than other STAT members (3–5 fold), indicating potential critical role of STAT3 in epilepsy (Fig. 3).

## Discussion

Vezzani et al. has well documented that CNS inflammatory processes may play crucial roles in the pathophysiology of seizures and epilepsy [23]. Cytokines are key mediators of both pro- and anti-inflammatory processes. Previous studies have implicated various cytokines in epilepsy [6–8, 24]. Here we tried to investigate the association of interictal concentrations of 10 cytokines to seizure severity in three types of epilepsy (TLE, XLE and IGE). Our data demonstrated 6 potential biomarkers and 3 independent biomarkers for severe epilepsy. No significant differences were observed among different types of epilepsy.

Prior publications have demonstrated the correlation between concentrations of certain cytokines and epilepsy. IL-6 is a pleiotropic cytokine expressed in various cell types and tissues [25]. Its serum concentration is elevated in the settings of many neurological disorders such as Alzheimer’s disease, trauma and meningitis [26–28]. Previous publications have indicated elevated IL-6 level after focal and generalized seizures in patients with epilepsy [26, 29–32]. In our study, serum interictal IL-6 concentration was the only one associated with all seizure severity evaluation scales in all three types of epilepsy. In multivariate analysis, it was positively linked to severity of all three types of epilepsy, indicating that serum IL-6 may be a good candidate to indicate seizure severity in general.

Cytokine levels in CSF are important markers for many different diseases [21]. IL-17a is involved in the development of many inflammatory diseases and has been reported to correlate with disease severity [33]. Another report indicated that interictal serum and CSF IL-17a concentrations were biomarkers for seizure severity [8], which is consistent to our findings. IL-17a is known to be able to facilitate migration of active T cells, including Th17, across the blood brain barrier [8]. Although, so far, there is no unequivocal proof that T cells exacerbate seizures directly, they may possibly play roles in the development of seizure indirectly, which could explain why IL-17a was significantly elevated in our study. This theory requires further investigation to proof.

IFNλs are novel cytokines and their roles in seizures have not been well defined yet. We evaluated the serum and CSF level of IFNλs and found that, among 4 IFNλs, IFNλ3 was associated with seizure severity and the time to next seizure episode (Tables 3, 4, 5), which was confirmed by mRNA concentration measurement (Fig. 2). This is consistent with previous findings in encephalitis [34].

Other cytokines in our study, such as IL-1b, IL-1Ra, and IL-8, were also correlated to seizure severity in univariate analysis (Table 3). IL-1b concentration was reported minimal changed postictally [6]. In our study, it was only correlated to VA score in XLE. Publications have shown that IL-1Ra level changed dramatically during seizure attack [6]. It was linked to seizure severity in all three types of epilepsy. As a pro-inflammatory chemokine, IL-8 has been reported to be involved in diseases of respiratory and neurological disorders [35–37]. In our study, IL-8 level was associated with seizure severity of all three types of epilepsy. This is consistent with previous publication [7].

## Conclusions

Among 14 cytokines, four independent biomarkers (IL-6, IFNγ, IL-17a and IFNλ3) for severe seizures in three different types of epilepsy were identified. All STATs were activated in epilepsy, among which, STAT3 was the most activated one. Important roles of these cytokines in the development of severe seizure could be speculated, and they may be used as potential markers to identify severe epilepsy.

## Abbreviations

CSF: cerebrospinal fluid
CNS: central nerve system
IFN: interferon
IL: interleukin
TLE: temporal lobe epilepsy
XLE: extra-temporal lobe epilepsy
IGE: idiopathic generalized epilepsy
NHS3: seizure severity scale 3
VA score: veterans administration seizures frequency and severity rating scale score
SD: standard deviation
